# Nosebleeds in Children as a Potential Marker for Nonaccidental Injury and Serious Underlying Pathology: How Aware Are Hospital Clinicians?

**DOI:** 10.5402/2011/909570

**Published:** 2011-09-26

**Authors:** Abdul Qader Ismail, Anjum Gandhi

**Affiliations:** Paediatric Department, Good Hope Hospital, Rectory Road, Sutton Coldfield, Birmingham B75 7RR, UK

## Abstract

Paediatric epistaxis is common and usually of benign origin. However, the differential diagnosis includes serious underlying pathology (e.g., bleeding disorders and blood cancers) and in the very young can be a marker of potential physical abuse. To assess if paediatric and A&E doctors were aware of the important differential, we asked them to complete a Likert scale questionnaire on several different clinical scenarios. Our results show that a significant proportion of doctors of all grades and in both specialties were either not aware of or not concerned about epistaxis in an infant as a possible sign of nonaccidental injury and were not willing to carry out simple blood tests to investigate recurrent nosebleeds in an older child. Our results highlight the need for education and evidence-based guidelines to avoid missing important, if infrequent, causes of paediatric epistaxis, both in the hospital and community setting.

## 1. Introduction

Epistaxis is common in children and often of benign origin. Occasionally, however, it can be due to serious underlying pathology and in the very young may be a marker for potential child abuse. Our aim was to determine if hospital clinicians expected to manage such patients are aware of the important differential diagnosis by asking them to complete a Likert scale questionnaire on several different clinical scenarios.

## 2. Paediatric Epistaxis

Nosebleeds in children are more common than in adults; reported in ~35% of 0–5 year olds, 56% of 6–10 year olds, and 64% of 11–15 year olds [1] compared to 10% of the general population [2].

The differential includes common but benign, as well as rare but serious, causes. In the former category are local (nose picking, trauma, and inflammation) and general causes (medications and environmental effects such as temperature and humidity). In the latter category, we have underlying pathologies including clotting disorders and cancer (e.g., hereditary haemorrhagic telangiectasia, idiopathic thrombocytopenic purpura (ITP), and leukemia/lymphoma) and nonaccidental injury (NAI).

## 3. Methods and Results

We asked A&E and paediatric doctors at our hospital (medium-sized DGH) to respond to a Likert scale questionnaire. In total, we had 39 completed questionnaires, 17 by paediatric and 22 by A & E doctors, with clinical experience ranging from 1.5 months to 33 years. The correct evidence-based answers are written in bold face (see Table 1). For every question, we received a variety of responses from doctors of all grades and in both specialties.

## 4. Discussion

As previously mentioned, an especially important differential in under-2 year olds is nonaccidental injury, since it is unusual for epistaxis to occur in this age group, with some studies showing that a significant proportion of such cases may be associated with child protection issues. McIntosh et al. [3] carried out a retrospective analysis of A&E department attendance records and hospital admissions for under-2 year olds and identified 16 cases over a 10-year period. Of these, 8 were associated with visible trauma and considered accidental at the time, 4 with thrombocytopenia of which 3 were due to underlying malignancy, and the other had ITP. Following review of the 16 cases, their history and presentation, it was decided by 3 independent paediatricians trained in child protection issues that in 50% of the cases child protection concerns should have been raised. Even studies which have found lower incidence rates of potential child abuse in their population studies stress that all cases of epistaxis in infants should have full and expert assessment to exclude bleeding disorders and physical abuse should be considered in this vulnerable age group especially if there are additional worrying features in history or clinical presentation [4].

Our results from question 1–4 show that up to nearly half of paediatric and A&E doctors were either not aware or not concerned about epistaxis in an infant as a possible sign of NAI.

When a child presents to hospital with epistaxis, especially with a history of previous such episodes, apart from treating the acute injury and blood loss, it is also an important screening opportunity for rare but important underlying pathologies mentioned above. This can be accomplished by a focused history (e.g., current medications, easy bruising, haemarthrosis, and leg pains), examination (e.g., telangiectasia on lips and mucous membranes, lymphadenopathy, and hepatosplenomegaly), and basic investigations (e.g., haemoglobin, white cell count, platelet count, and clotting studies). 

In the majority of cases, no underlying cause will be found. Most episodes of idiopathic epistaxis are self-limiting [5]. Where a cause can be identified, nose-picking is most common. Furthermore, the majority of paediatric nosebleeds are dealt with in community, either by the patient, relatives, or their school [6]; this is supported by our findings that 14 out of 39 paediatric and A&E doctors, with up to 6 years of experience, had not encountered paediatric epistaxis. However, by carrying out these simple steps, important pathology will not be missed, and, in such cases, further investigation and treatment can be instigated.

Questions 5 and 6 both test whether the doctor was aware of serious underlying pathology as a differential. While a majority of the doctors seem to have this knowledge, significantly fewer doctors were willing to carry out simple blood tests which would provide more objective, if not arguably more valuable information than the history or examination (~50% versus ~85%), even though the child in the scenario had recurrent nosebleeds.

Verbal feedback that we received indicates that this disparity is mainly due to indecision about whether such tests are warranted to find a very uncommon cause for such a “minor,” self-resolving problem, especially when the parent(s) may be eager to take their child home and would then have to wait an hour or more for results.

## 5. Conclusion

Our results highlight the need for education and evidence-based guidelines to avoid missing important, if infrequent, differential diagnoses of paediatric epistaxis. Furthermore, given that the majority of cases are self-limiting and dealt with in the community, it is of potentially greater importance that primary care healthcare staff (i.e., GPs and health care visitors) are aware that nosebleeds are uncommon in children under 2 and may indicate nonaccidental injury. Interestingly, the GP Notebook which is the commonly used educational resource in primary care does not highlight this point sufficiently and perhaps needs updating [7]. 

## Figures and Tables

**Table 1:** Likert scale questionnaire and results.

Questions		“Strongly Agree” or “Agree”	“Neither Agree nor Disagree”	“Disagree” or “Strongly Disagree”
(1)	A 6-month-old boy is brought in by his mother with a nosebleed. Just as you are reassuring the mother your colleague pulls you to a corner and states that you need to be very careful with your clinical assessment as epistaxis in a child of this age can be a sign of child abuse. Do you:	**24**	8	7

(2)	He suggests that your history should include a thorough questioning of how this event occurred, any previous such events, and any previous injuries the child may have sustained, including hospital admissions. If there is any doubt whatsoever, you should be ready to seek senior help and make enquiries from social services and mention your concerns. Do you:	**35**	2	2

(3)	The mother of the child tells you that the baby was asleep in his cot, and when she heard him crying on the baby monitor and went to fetch him, she noticed the bleeding nose. The nurse working with you says that this kind of spontaneous nose bleed is not uncommon in babies and starts reassuring the mother. Do you:	7	10	**22**

(4)	You discuss the child with an ENT colleague on the ward. He tells you that epistaxis is quite common in children and laughs at you when mention that you are concerned about physical abuse. Do you:	3	7	**29**

(5)	The next day a 9-year-old girl presents with a nosebleed. On taking the history you learn she has had two previous such episodes earlier this year. The nosebleed started spontaneously, resolved with first aid measures, and the girl is haemodynamically stable. Her mother is eager to take her home. Just as you are about to discharge them, your colleague asks if you have taken blood to check Hb levels, platelet count, and INR. Do you:	**20**	4	15

(6)	He also asks if your history involved questions to inquire about possible underlying causes such as hereditary haemorrhagic telangiectasia, idiopathic thrombocytopenic purpura, leukemia/lymphoma, and haemophilia. Do you:	**33**	1	5
